# The effect of pituitary neuroendocrine tumors on the volumes of intracranial structures

**DOI:** 10.3389/fneur.2025.1585921

**Published:** 2025-09-10

**Authors:** Abdulkerim Gökoğlu, Hüseyin Yiğit, Ebru Yolaçan, Mehtap Nisari, Erdoğan Unur, Ahmet Selçuklu

**Affiliations:** ^1^Department of Neurosurgery, Dünyam Hospital, Kayseri, Türkiye; ^2^Vocational Health School, Cappadocia University, Nevşehir, Türkiye; ^3^Department of Anatomy, Faculty of Medicine, Samsun University, Samsun, Türkiye; ^4^Department of Anatomy, Faculty of Medicine, Erciyes University, Kayseri, Türkiye

**Keywords:** intracranial volume, neuroendocrine surgery, neuronavigation, pituitary neuroendocrine tumors, Vol2brain

## Abstract

**Objective:**

Pituitary Neuroendocrine Tumors (PitNETs) can cause symptoms via mass effect or hormonal imbalances. This study investigated whether PitNETs induce volumetric changes in intracranial structures and assessed the diagnostic potential of these changes.

**Materials and methods:**

A retrospective analysis was conducted on 90 PitNET patients and 86 healthy controls. MRI data, acquired on a 1.5 Tesla scanner, were processed using the automated Vol2Brain system to calculate relative brain volumes.

**Results:**

PitNET patients exhibited significantly lower relative volumes across numerous brain structures compared to controls. This included reduced intracranial, cerebral, and cortical gray matter (GM), as well as temporal lobe, vermis, limbic lobe, hippocampus, and inferior lateral ventricle (ILV) volumes. Gyrus-level analysis also revealed significantly smaller volumes in key regions like the posterior orbital gyrus, supplementary motor cortex, and entorhinal area in PitNET patients. ROC analysis demonstrated good to very good diagnostic performance for ILV volume (AUC = 0.863; *p* = 0.002) and subcortical GM volume (AUC = 0.725; *p* = 0.049) in differentiating groups. Reduced volumes were also noted in basal ganglia structures.

**Conclusion:**

Our findings indicate significant volumetric reductions in various brain regions in PitNET patients, potentially explaining observed emotional and cognitive symptoms. The diagnostic utility of ILV and subcortical GM volumes is promising, suggesting their value as diagnostic adjuncts. These objective volumetric assessments may assist in surgical planning and patient stratification, though further prospective research is warranted to establish direct links with clinical outcomes.

## Introduction

As the master regulator of numerous physiological processes, the pituitary gland controls downstream endocrine glands (1). Pituitary Neuroendocrine Tumors (PitNETs) are common sellar tumors that can cause symptoms either through hormonal effects or due to mass effect on the stalk and/or the gland when they are large (2). PitNETs are benign tumors that are the third most common intracranial tumors after meningiomas and diffuse glial tumors. The incidences of these tumors are 4.36 per 100,000 and can affect all age groups (3). Pituitary tumors are classified in the 2022 5th edition WHO classification of Endocrine and Neuroendocrine Tumors (ENDO5) (4). According to the ENDO and CNS5 grading methods, pituitary adenomas are graded based on radiological imaging findings, mitosis, invasion and Ki67 levels (5). There is also a different method of estimating pituatory tumor risks known as the clinicopathological classification of Trouillas (6, 7). According to this system, tumors are graded based on invasion, proliferative activity (Ki-67, mitotic index), and p53 positivity.

It is crucial to acknowledge that depression, anxiety, and cognitive dysfunction are frequently observed comorbidities in patients diagnosed with PitNETs. These disorders can significantly impair a patient’s quality of life and may have a substantial impact on their overall prognosis (8).

Lang et al. investigated the relationship between visual outcomes and functional connectivity (FC) in patients who underwent surgery for pituitary adenomas. The study involved 21 patients with pituitary tumors and 19 healthy controls. The authors found that increased FC of the visual cortex was associated with good visual outcomes in patients with pituitary tumors. They also found that resting-state functional MRI (rsfMRI) can distinguish between patients with good and poor visual outcomes after pituitary tumor surgery. The authors suggest that rsfMRI may have a future role in characterizing the impact of cortical adaptation on visual recovery (9).

Nakaya et al. developed a novel method, termed semiautomatic segmentation with manual adjustments (SSMA), for volumetric measurement of the nasal cavity and paranasal sinuses, a crucial step in planning endoscopic endonasal surgery for pituitary tumors. The researchers demonstrated the accuracy and efficiency of SSMA, and its ability to predict the extent of tumor removal in patients with non-functioning pituitary tumors invading the cavernous sinus (10).

In a study conducted by Tang et al., the researchers investigated the microstructural alterations induced by PitNETs and gliomas within the brain. Their findings revealed that PitNETs exert a comparatively mild and non-invasive impact on brain tissue, primarily influencing neural function through the aberrant secretion of hormones or by imposing compression on adjacent cerebral structures. Conversely, gliomas were observed to instigate destructive infiltration of cortical and subcortical regions, thereby precipitating more pronounced cognitive impairments (11).

The existing literature presents a paucity of information regarding the potential of PitNETs to induce volumetric alterations in intracranial structures. We hypothesize that the compressive forces exerted by PitNETs on adjacent brain tissues may precipitate volumetric changes, particularly within structures proximal to the pituitary gland. Consequently, the primary objective of this study is to investigate the potential of PitNETs to elicit volumetric changes in intracranial structures.

## Materials and methods

### Patients

For this retrospective study, 90 patients with PitNET and 86 healthy participants without central nervous system (CNS) disease, with age and gender distribution similar to the patient group were enrolled. Patients with known PitNETs, who had not undergone any previous surgical intervention for any purpose and had no known central or peripheral nervous system disorders, were included in the retrospective analyses. All patients were pituitary adenomas without previous surgical or radiotherapy treatment. Approval was obtained from the Local Ethics Committee before the start of the study. All procedures were carried out in accordance with the 1964 Helsinki Declaration and its later amendments. Written informed consent was obtained from all participants.

### Radiological imaging

#### MR protocols

In our study, 1.5 Tesla MRI Aera scanner (Siemens, Germany) with clinical field strength were used in all patients using the routine pituitary protocol. The images were acquired using a 3D magnetization-prepared rapid acquisition gradient echo (MPRAGE) imaging sequence. The nominal parameters for MPRAGE were as follows: sagittal plane, TR/TE/TI 2,400/3/1,000 ms, flip angle 8°, 24 cm FOV, 192 × 192 in-plane matrix, 1.2 mm section thickness.

#### Volumetric analysis

**Figure 1:**
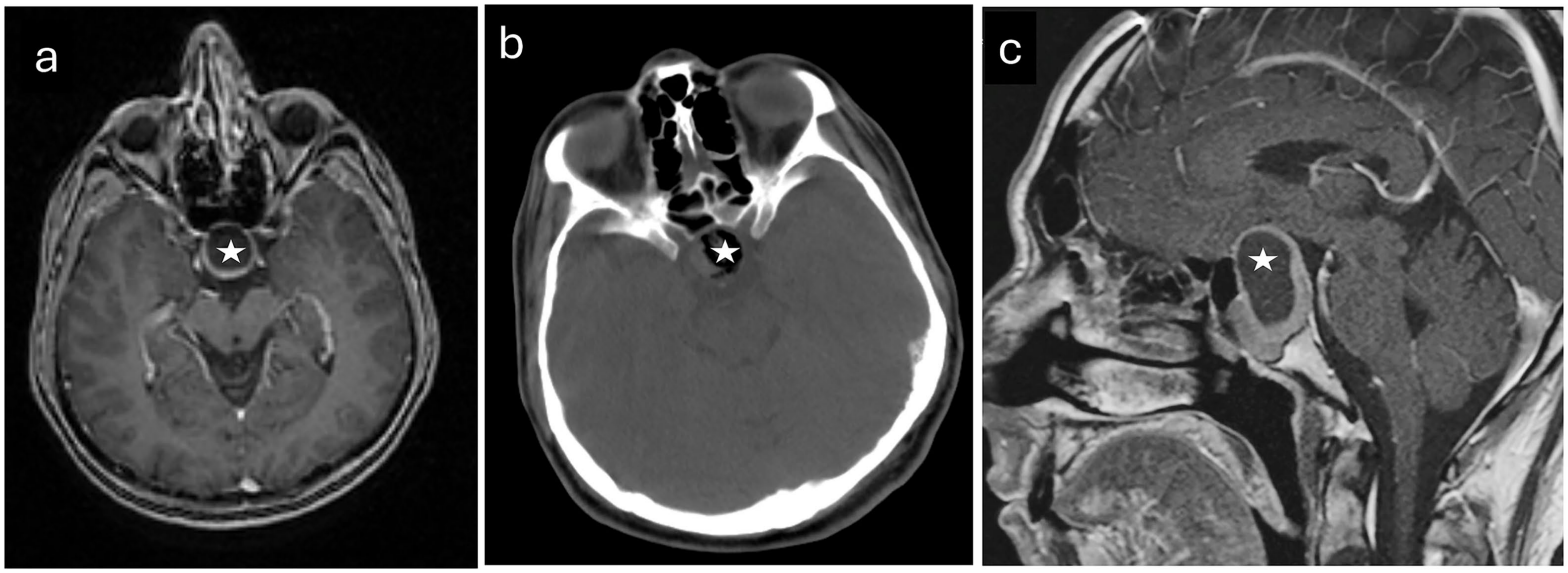
Radiological images pertaining to a representative patient diagnosed with pituitary neuroendocrine tumor who participated in the study. **(a)** An axial T1-weighted magnetic resonance image acquired prior to surgical intervention. **(b)** A computed tomography scan obtained before the operation. **(c)** A sagittal MR image taken preoperatively. The tumors are identified by white stars.

All MRI data was processed in a virtual environment. A sample radiological image included in the study is shown in Figure 1. To detect volumetric differences, MRIs of 86 control subjects without CNS disorders and 90 patients with PitNET were obtained through archival scanning. MRI data were converted to DICOM using Radiant-DICOM-Viewer software.^1^ Image files in sagittal 3DT1 DICOM (Digital Imaging and Communications in Medicine) format for all participants were converted to NIFTI-1 (Neuroimaging Informatics Technology Initiative) format and uploaded to the vol2Brain system.

Automatic and reliable quantitative tools for MRI brain image analysis have become an invaluable resource for clinical use and research settings. The past few decades have seen many advances with successful techniques based on deep learning. Vol2Brain is an online MRI brain volume measurement method that processes MRI data to calculate local concentration differences of brain tissues using Vol2Brain. Vol2Brain is free software, provides fast results, and does not require additional procedures such as installation and adaptation. Vol2Brain is currently available through the existing volBrain platform (12).^2^ Vol2Brain is a fully automated segmentation technique based on a multi-atlas patch-based label fusion segmentation technology. The Vol2Brain workflow is based on the following steps: (i) Preprocessing, (ii) Multiscale labeling and cortical thickness estimation, (iii) Reporting and CSV generation. All volumes were presented as absolute values (measured in cm^3^) and relative values (measured according to ICV). The Asymmetry Index was calculated by dividing the difference between the right and left volumes by their averages (as a percentage: %). The segmentation images were in MNI space (neurological orientation). For the current analysis, the subvolumes of cerebrospinal fluid (CSF), total brain, infratentorial brain, supratentorial brain, total white matter (WM), total gray matter (GM), cortical GM, and subcortical GM (putamen, nucleus caudatus, amygdala, globus pallidus, ventral DC, thalamus, etc.) were also automatically measured (13, 14).

To prevent bias in the comparison between the patient and control groups and to ensure reader blinding, the image interpretation and collection and analysis of demographic data were conducted separately. Patients were labeled with pseudonyms and recorded in separate tables.

### Statistical analysis

Statistical analyses were conducted using SPSS 26.0 (IBM Corporation, Armonk, New York, United States) and GraphPad Prism 9.0. Data normality was assessed via the Kolmogorov–Smirnov test, and homogeneity of variance was evaluated using Levene’s test. Quantitative variables between patient and control groups were compared using either the Independent-Samples T-test with Bootstrap results or the Mann–Whitney U test with Monte Carlo results, as appropriate. Gender comparisons between groups were performed using Fisher’s Exact test with Monte Carlo simulation. Receiver Operating Characteristic (ROC) curve analysis was employed to determine the sensitivity, specificity, positive predictivity, and negative predictivity of Subcortical Gray Matter and Inferior Lateral Ventricle (ILV) variables, based on their respective cutoff values, for group classification. All analyses were conducted at a 95% confidence level, with a *p*-value less than 0.05 considered statistically significant (*p < 0.05, **p < 0.01, ***p < 0.001, ****p < 0.0001).

## Results

The demographic characteristics of individuals with PitNETs and healthy controls are presented in Table 1. Among the 90 individuals with PitNETs (*n* = 90) whose retrospective data were screened, 27.7% (*n* = 25) were female, while 72.3% (*n* = 65) were male. In the control group, the proportion of females and males among the 86 healthy individuals (*n* = 86) was equal (*n* = 43, 50% each, respectively). The mean age in the PitNET group was 43.2 years, whereas it was 37.2 years in the healthy control group. PitNETs are recognized to exhibit a higher prevalence in females compared to males (15). However, we observed a predominance of male patients with PitNETs presenting at our study. Furthermore, age is also known to influence brain volume (16). While the mean ages of our patient and control groups were comparable, they were not perfectly matched. To control for the potential confounding effects of sex and age on the volumetric analysis of intracranial structures affected by PitNETs, we utilized relative volume (cm^3^/total intracranial volume) measurements in our analyses, rather than absolute volumes (cm^3^).

**Table 1:** Demographic findings of the healthy control group and pituitary neuroendocrine tumor patients.

	Total	Control	Patient
	(*n* = 176)	(*n* = 86)	(*n* = 90)
	Mean (SD) (min-max)	mean (SD) (min-max)	Mean (SD) (min-max)
Age	40.3 (14.8) (16–82)	37.2 (14.3) (21–55)	43.2 (21.1) (16–82)
Gender	n (%)	n (%)	n (%)
Female	68 (38.6)	43 (50.0)	25 (27.7)
Male	108 (61.4)	43 (50.0)	65 (72.3)

Table 2 presents a comparative analysis of hormonal profiles in individuals with PitNETs before and after surgical intervention. This evaluation encompasses key hormonal axes, including thyroid hormones (fT3, fT4, TSH), reproductive hormones (total testosterone, prolactin, FSH, LH, E2), growth hormone (GH and Somatomedin C), and stress hormones (ACTH and cortisol). While a slight decrease in fT3 and fT4 levels was observed postoperatively, this change was not statistically significant. Similarly, TSH levels increased slightly after surgery, but this increase was also not statistically significant. Regarding reproductive hormones, a slight decrease in total testosterone levels was noted postoperatively, while an increase in prolactin levels was evident. FSH and LH levels also showed postoperative increases, with a particularly pronounced increase in LH. However, no significant change was observed in E2 levels. These findings indicate that PitNET surgery may have complex effects on the pituitary-gonadal axis, potentially leading to alterations in gonadal function in some patients. A significant decrease in Somatomedin C levels was observed postoperatively, accompanied by a notable increase in GH levels. This suggests that surgical intervention may influence GH secretion, with potential implications for growth and metabolism. Finally, a slight decrease in ACTH and cortisol levels was observed postoperatively, but these changes were not statistically significant. This suggests that PitNET surgery has minimal impact on the hypothalamic–pituitary–adrenal axis.

**Table 2:** Pre- and post-operative hormonal profiles of individuals with PitNETs.

Hormone	Preoperative	Postoperative
	Mean±SD	Mean±SD
fT3 (ng/dl)	2,89 ± 0,66	2,14 ± 0,31
fT4 (ng/dl)	2,53 ± 3,51	2,28 ± 3,29
TSH (mIU/L)	1,61 ± 0,90	1,72 ± 2,28
Total Testosteron (ng/dl)	2,15 ± 2,20	1,79 ± 1,11
Prolactin (μg/L)	12,28 ± 11,19	21,38 ± 24,35
Somatomedin C (pmol/L)	175,74 ± 108,54	131,52 ± 24,96
FSH (mIU/L)	2,89 ± 1,77	3,98 ± 2,39
LH (mIU/L)	1,95 ± 0,92	14,8 ± 31,88
E2 (pg/ml)	16,99 ± 7,69	19,58 ± 11,08
GH (mcg/L)	0,15 ± 0,04	14,74 ± 3,09
ACTH (pg/ml)	21,92 ± 12,34	19,8 ± 13,21
Cortisol (mcg/dL)	11,12 ± 8,96	9,3 ± 1,99

fT, free thyroxine; TSH, Thyroid Stimulant Hormone; FSH, Follicle Stimulant Hormone; LH, Luteinizan Hormone; E2: Estradiol; GH, Growth Hormone; ACTH, Adrenocorticotrop Hormone; SD., Standard deviation.

**Figure 2:**
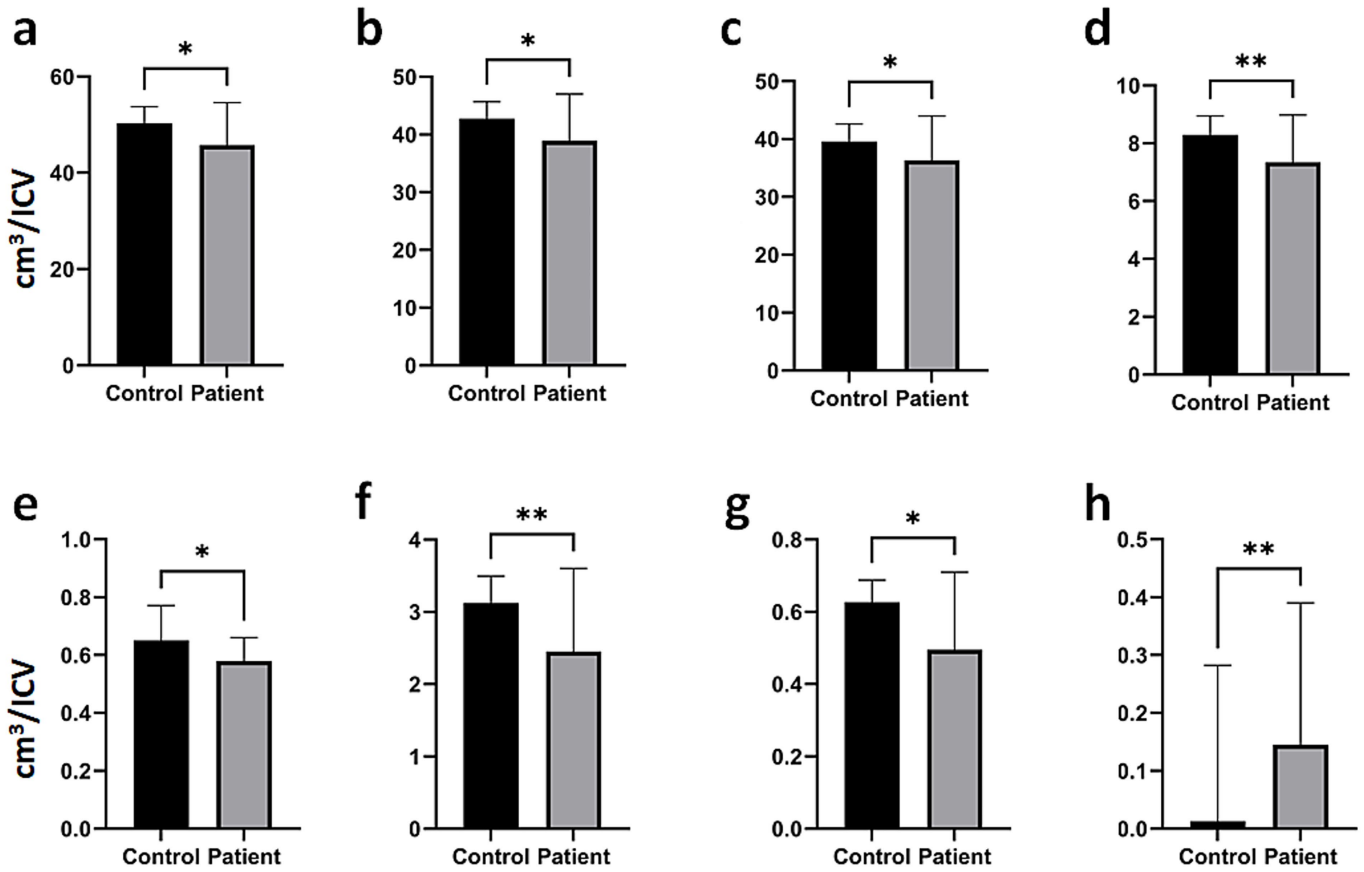
Comparison of major brain structures between PitNET patients and healthy controls. **(a)** Total intracranial GM, **(b)** total cerebral GM, **(c)** total Cortical GM, **(d)** total temporal volume, **(e)** vermis, **(f)** total limbic lobe, **(g)** total hippocampus volume, **(h)** total inferior lateral ventricle. Bar plots represent the median and range. Significant differences between groups were analyzed using a two-tailed Mann–Whitney U test (*p < 0.05, **p < 0.01).

The analysis results for major brain structures showing significant differences between individuals with PitNETs and healthy controls are presented in Figure 2. The intracranial total GM volume (Figure 2a; *p < 0.05), cerebral GM volume (Figure 2b; *p < 0.05), cortical GM volume (Figure 2c; *p < 0.05), temporal lobe volume (Figure 2d; **p < 0.01), vermis volume (Figure 2e; *p < 0.05), limbic lobe volume (Figure 2f; **p < 0.01), hippocampus volume (Figure 2g; *p < 0.05), and inferior lateral ventricle volume (Figure 2h; **p < 0.01) were significantly lower in the PitNET group compared to the healthy control group.

**Figure 3:**
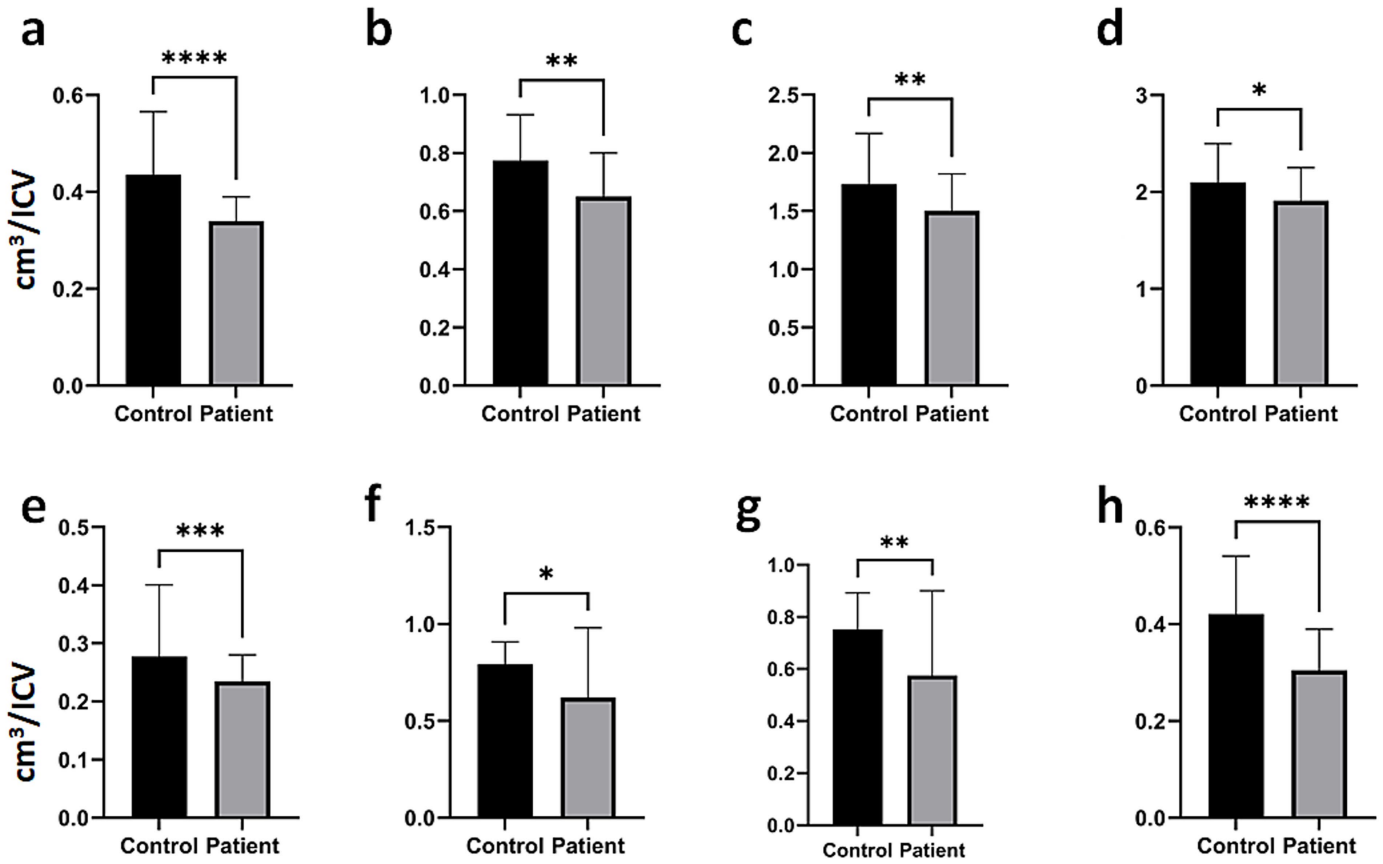
Gyrus analysis results demonstrating significant differences between patients with PitNETs and healthy controls. **(a)** posterior orbital gyrus, **(b)** supplementary motor cortex, **(c)** inferior temporal gyrus, **(d)** medial temporal gyrus, **(e)** entorhinal area, **(f)** middle cingulate gyrus, **(g)** posterior cingulate gyrus, and **(h)** parahippocampal gyrus. Bar plots illustrate the median values and their respective ranges. Statistical significance between the groups was determined using a two-tailed Mann–Whitney U test (*p < 0.05, **p < 0.01, ***p < 0.001, ****p < 0.0001).

Analysis results demonstrating significant differences in gyrus-level brain structures between individuals with PitNETs and the healthy control group are presented in Figure 3. Individuals with PitNETs exhibited significantly smaller volumes in the posterior orbital gyrus (Figure 3a; ****p < 0.0001), supplementary motor cortex (Figure 3b; **p < 0.01), inferior temporal gyrus (Figure 3c; **p < 0.01), medial temporal gyrus (Figure 3d; *p < 0.05), entorhinal area (Figure 3e; ***p < 0.001), middle cingulate gyrus (Figure 3f; *p < 0.05), posterior cingulate gyrus (Figure 3g; **p < 0.01), and parrahippocampal gyrus (Figure 3h; ****p < 0,0001) compared to healthy control individuals. Furthermore, the analysis results for total (right and left hemispheres combined) brain structures, structures located in the right hemisphere, and structures located in the left hemisphere are presented in Supplementary Figures 1–3, respectively.

**Figure 4:**
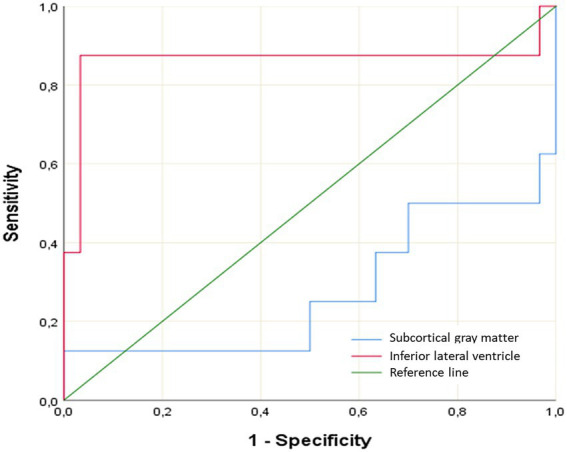
ROC curve for subcortical GM volume and inferior lateral ventricular volume.

ROC analysis was performed to detect differences in “Subcortical GM” and “Inferior Lateral Ventricular Volume” between groups (Table 3). For “Subcortical GM,” when the cutoff value was set at 3.0361, sensitivity was found to be 63.5% and specificity was 63.3% (AUC ± Std: 0.725 ± 0.122; *p* = 0.049). For “Inferior Lateral Ventricular Volume,” when the cutoff value was set at 0.0465, sensitivity was determined to be 87.5% and specificity was 90.7% (AUC ± Std: 0.863 ± 0.112; *p* = 0.002; Figure 4).

**Table 3:** ROC curve analysis for subcortical GM and inferior lateral ventricular volume.

	Subcortical gray matter	Inferior lateral ventricle
Cut-Off	3.0361	0.0465
Sensitivity	0.635	0.875
Specificity	0.633	0.907
+PV	63	90
–PV	63	88
AUC (Std)	0.725 (0.122)	0.863 (0.112)
*p* value	0.049	0.002

ROC, Receiver Operating Curve Analysis; AUC, Area under the ROC curve; PV, Predictive Value; Std, Standard Deviation.

## Discussion

Pituitary macroadenomas can extend into the suprasellar region, exerting pressure on the optic nerves and chiasm. Visual impairment is observed in approximately 40–60% of patients at the time of presentation, with classical presentation being bitemporal hemianopsia. Additionally, growth of the suprasellar mass can cause hypothalamic compression, leading to disorders in eating, emotional, or sleep patterns (17–20). Rarely, enlargement of the third ventricle can lead to obstructive hydrocephalus. Parasellar expansion into the cavernous sinus is usually asymptomatic, but pressure symptoms may arise in the oculomotor, abducens, and trigeminal nerves (21, 22). Additional parasellar expansion can compress the mesial temporal lobe, potentially leading to seizures. However, determining the true relationship between pituitary damage and headaches is often difficult (23). Patients with functioning PitNET are often diagnosed before patients with non-secretory PitNET due to early symptomatology. The development of symptoms in these patients is thought to be related to the effect of mass compression on adjacent structures and, indirectly, to volume changes in intracranial structures. In our study, individuals with PitNETs were found to have significantly lower volumetric values in various brain structures compared to the control group. These findings may help explain some of the symptoms observed in patients. Notably, the volumetric reduction in intracranial (Figure 2a), cerebral (Figure 2b), and cortical GM (Figure 2c) in PitNET patients was prominent, particularly in brain regions responsible for cognitive and motor functions. Severe volumetric changes were observed especially in structures adjacent to the PitNET’s location (Figures 2e,g, 3a,c,d). Furthermore, considering the limbic system’s control over emotional behaviors (24), the volumetric decrease in total limbic lobe volume (Figure 2f), hippocampus volume (Figure 2g), entorhinal area volume (Figure 3e), middle cingulate gyrus volume (Figure 3f), posterior and anterior cingulate gyrus (Figures 3f,g, respectively) and parahippocampal gyrus (Figure 3h) in PitNET patients is remarkable. As previously mentioned, the occurrence of emotional changes in patients is consistent with volumetric alterations in limbic structures.

The inferior lateral ventricle, a cerebrospinal fluid-filled cavity nestled deep within the brain, boasts a fascinating array of neighboring structures. Its floor, in particular, lies in close proximity to several key components of the brain. One of the most prominent neighbors is the hippocampus, a crucial structure for memory and learning, and a key part of the limbic system. The hippocampus forms the medial wall of the ventricle. Nearby lies the amygdala, a structure known for its role in emotional responses, especially fear. Emanating from the hippocampus, the fimbria fibers, which form part of the fornix, connect the hippocampus to the mammillary bodies. The parahippocampal gyrus, involved in memory and spatial navigation, also resides in this region, located on the medial surface of the temporal lobe. The choroid plexus, responsible for producing cerebrospinal fluid, is located in the floor of the ventricle. The lateral wall houses the thalamus, a critical relay center for sensory and motor information. Also nestled within the lateral wall is the caudate nucleus, a component of the basal ganglia involved in motor control, learning, and memory. Another basal ganglia structure, the globus pallidus, which plays a role in motor control, is found in the same vicinity. Lastly, the optic radiations, fibers carrying visual information from the thalamus to the visual cortex, traverse the lateral wall of the temporal horn. This intricate network of neighboring structures highlights the clinical significance of the inferior lateral ventricle. Any pathology affecting this region, such as a tumor, hemorrhage, or hydrocephalus, can have far-reaching consequences for the surrounding structures (25, 26). Therefore, a thorough understanding of the anatomy of the inferior lateral ventricle is crucial for the diagnosis and treatment of neurological diseases. The study conducted by Ge et al. provides compelling evidence for a strong association between increased inferior lateral ventricle volume and Alzheimer’s disease (27). Arakaki et al. conducted a study on 136 individuals, demonstrating an inverse relationship between age and hippocampal volume. Their findings revealed that as hippocampal volume decreased with age, inferior lateral ventricle volume increased. Furthermore, they correlated these structural changes with declines in cognitive function (28). In our study, PitNET patients were found to have significantly smaller volumes in the basal ganglia, including the amygdala, caudate nucleus, and basal forebrain (total, right, and left), as well as in the right putamen, compared to the control group (Supplementary Figures 1–3). The notable reduction in ILV volume in PitNET patients relative to healthy individuals (Figure 2h; Supplementary Figures 2, 3), in addition to the volumetric change in the left medial temporal gyrus, may be associated with the involvement of the post-optic chiasm visual pathways in these patients. However, further studies are required to confirm this association. Hormones are known to be linked with dementia (29–31). Furthermore, it is being investigated whether PitNET also causes hormonal imbalances in patients (32). The volumetric changes observed in our study in regions closely associated with memory (temporal and hippocampal structures) indicate the need for advanced studies examining dementia, PitNET, and brain structures in conjunction.

The development of symptomatic hydrocephalus due to a PitNET is an exceptional event. PitNET can lead to different symptoms due to their proximity to optical structures, cisterns, and brain parenchyma. PitNET may rarely cause obstructive hydrocephalus due to mass effect and enlargement of the third ventricle. In our study, it was found that the pituitary tumor only had significant differences in the volume of the inferior ventricle.

Tirosh et al. showed that 3D volume measurements correlated better with initial surgical success rates and disease control compared to standard measurements in patients with GH-secreting PitNET. Furthermore, 3D volume measurements were shown to be better predictors of postsurgical outcomes for anterior pituitary tumors in general (33).

As a nonfunctional pituitary adenomas (NFPA) grows, the tumor exerts a mass effect on surrounding structures which can induce focal neurological deficits such as an impaired visual field or visual acuity and even blindness (34). There have been a number of studies investigating tumor volume calculations, but many of these studies have focused on the limitations of traditional methods when assessing pituitary tumor resections, particularly in cases involving irregularly shaped tumors such as Cushing’s disease, acromegaly, and/or significant cavernous sinus invasions (35, 36). There are few studies evaluating the proportional volumetric changes of pituitary adenoma and intracranial structures (17, 37).

It is also important to assess volumetric progression of incidentalomas, especially microadenomas, in order to determine surgical indications (38). However, in our study, intracranial volumetric variables were evaluated especially in patients who underwent surgery for NFPA. To our knowledge, no similar volumetric studies focusing on this specific patient population and context have been reported in the literature (34).

Our study also evaluated preoperative and postoperative hormonal profiles in patients with PitNET. Significant changes were observed, particularly in growth hormone and somatomedin C levels, alongside variations in other hormonal parameters such as TSH, prolactin, and testosterone. These findings emphasize the impact of pituitary adenoma resection not only on the mass effect and volumetric changes but also on the hormonal milieu (39). The alterations in hormonal levels underline the importance of comprehensive perioperative hormonal monitoring and management to ensure optimal postoperative outcomes.

Our study has some limitations. First, the study cohort was limited to adults with memory complaints living in the community, which may not represent the general elderly population. Second, an imaging protocol was used on a 1.5 Tesla MRI scanner with a section thickness (1.2 mm) larger than the standard used for anatomical analyses (1 mm) (40). Third, although we were able to control for significant features that could affect the relationship, there may still be residual potential confounders. Fourth, our study is currently cross-sectional, which limits conclusions regarding causality. Another limitation of our study was the ratio of male and female patients was not equal. As is known, the frequency of PitNET in women is slightly higher than in men, but it shows a heterogeneous distribution pattern based on gender. Therefore, relative volume was used instead of absolute volume in this study. The strengths of our study include data collection from a same scanner and same research center, rigorous MR imaging and assessment methodology, automated and reliable free software, Vol2Brain, which provides more detailed results. Finally, with the current information, we cannot definitively explain whether the observed volumetric reduction in brain structures in PitNET patients compared to the control group is due to edema, vascular congestion, or tissue remodeling. Therefore, future studies are needed to investigate the mechanisms by which the mass effect of the tumor leads to such volumetric decreases.

ILV volume represents a significant neurological indicator susceptible to various influencing factors. Conditions such as increased intracranial pressure (41), hydrocephalus (42), normal pressure hydrocephalus, and Alzheimer’s disease (43) can induce alterations in the inferior lateral ventricle volume. Early detection and management of these conditions are crucial for preventing neurological damage and enhancing patient quality of life. Our study revealed a significant increase in ILV volume in patients with PitNETs compared to the healthy control group. Considering the anatomical relationships of the ILV, it is plausible that there is a link between the observed volumetric increase in the ILV and the patients’ reported forgetfulness (data not shown). However, further studies are needed to confirm this association and to evaluate the postoperative outcomes of PitNET patients, including a comparison of their forgetfulness before and after surgery.

Increased ILV and subcortical GM volumes may potentially enhance the precision and effectiveness of surgical resection by improving the preoperative understanding of tumor margins, which is particularly crucial when aiming for total resection. Preoperative volumetric analysis can contribute to determining the optimal surgical approach, such as transsphenoidal or craniotomy, by evaluating the tumor’s impact on intracranial structures. Furthermore, preoperative volumetric assessment can guide the determination of the optimal sellar base craniectomy extent and location, thereby enhancing the safety and efficacy of surgical access. This information can particularly assist surgeons in performing a more controlled and safer craniectomy, especially in cases of wide-based tumors or anatomical variations.

The cutoff values and ROC analysis results identified in our study suggest the potential of ILV volume as valuable diagnostic adjuncts in the diagnosis of PitNETs. This may be particularly beneficial in challenging differential diagnosis scenarios or in early stages when clinical suspicion is high. However, it is essential to validate the diagnostic accuracy of these findings in larger cohorts and diverse clinical settings. It should be emphasized that these volumetric measurements are intended to support and strengthen, rather than replace, routine diagnostic algorithms.

Preoperative volumetric analysis offers a means of objectively assessing the ‘mass effect’ of PitNETs on intracranial structures. This information can potentially be used to stratify patients in terms of their risk of developing symptoms or their prognosis. Nevertheless, further prospective studies are needed to establish a direct relationship between volumetric changes and symptom severity or disease prognosis. While our current study demonstrates the presence of these volumetric alterations, it does not fully elucidate their correlation with clinical outcomes.

## Conclusion

This study revealed significant volumetric alterations, specifically decreases in total GM, cerebral GM, cortical GM, temporal lobe, vermis, limbic lobe, and hippocampus, alongside a notable increase in inferior lateral ventricle (ILV) volume, in patients with Pituitary Neuroendocrine Tumors (PitNETs). These changes were prominent in regions proximal to the tumor and within structures critical for cognitive and emotional functions.

The observed volumetric reductions in limbic structures provide an anatomical correlate for the frequently encountered emotional and cognitive impairments in PitNET patients. Conversely, the significant increase in ILV volume emerges as a potential biomarker for PitNET-related neurological sequelae. The sensitivity and specificity of ILV volume, as determined by ROC analysis, underscore its value as a diagnostic adjunct.

Post-surgical hormonal changes further indicate the tumor’s intricate structural and functional interactions. Our findings emphasize that the widespread brain volumetric changes in PitNET patients contribute to their diverse clinical presentations, highlighting the importance of comprehensive neurological and psychological evaluations. Further prospective studies are warranted to establish the direct relationship between ILV volume and clinical outcomes, and to ascertain its utility in surgical planning.

## Data Availability

The datasets presented in this article are not readily available because the data that support the findings of this study are available from the corresponding author, upon reasonable request. Requests to access the datasets should be directed to Abdulkerim Gökoğlu, akerimg@hotmail.com.

## Footnotes

^1^ https://www.radiantviewer.com

^2^ https://volbrain.upv.es

## Supplementary material

SUPPLEMENTARY FIGURE 1 Analysis of total basal nuclei and cerebellar gray matter volumes. *Acc*, Nucleus accumbens, *Amy*, Amygdala, *BasF*, Basal forebrain, *Cau*, Nucleus caudatus, and *CerebelGM*, Cerebellar gray matter volume. Bar plots illustrate the median values and their respective ranges. Statistical significance between the groups was determined using a two-tailed Mann-Whitney U test (**p*<0.05, ***p*<0.01, ****p*<0.001, *****p*<0.0001).

SUPPLEMENTARY FIGURE 2 Volumetric difference analysis results of structures located in the left hemisphere between PitNET and healthy control groups. *Acc*, Nucleus accumbens, *Amy*, Amygdala, *BasF*, Basal forebrain, *Cau*, Nucleus caudatus, *Enth*, Entorhinal area, *Hip*, Hippocampus, *iLV*, Inferior lateral ventricle, *ITG*, Inferior temporal gyrus, *Lim*, Limbic lobe, *OCP*, Occipital pole, *PCgG*, Posterior cingulate gyrus, *PHG*, Parahippocampal gyrus, *POrG*, Posterior orbital gyrus, *SMC*, Supplementary motor cortex, *TL*, Temporal lobe. Bar plots illustrate the median values and their respective ranges. Statistical significance between the groups was determined using a two-tailed Mann-Whitney U test (**p*<0.05, ***p*<0.01, ****p*<0.001, *****p*<0.0001).

SUPPLEMENTARY FIGURE 3 Volumetric difference analysis results of structures located in the left hemisphere between PitNET and healthy control groups. *Acc*, Nucleus accumbens, *Amy*, Amygdala, *BasF*, Basal forebrain, *CerebelGM*, Cerebellar GM volume, *Cerebellum*, Left Cerebellum Hemisphere, *Enth*, Entorhinal area, *Hip*, Hippocampus, *iLV*, Inferior lateral ventricle, *ITG*, Inferior temporal gyrus, *Lim*, Limbic lobe, *MCG*, Middle cingulate gyrus, *MTG*, Middle temporal gyrus, *OCP*, Occipital pole, *PCgG*, Posterior cingulate gyrus, *PHG*, Parahippocampal gyrus, *POrG*, Posterior orbital gyrus, *Put*, Putamen, *SMC*, Supplementary motor cortex, *SPL*, Superior Parietal Lobule, *TL*, Temporal lobe. Bar plots illustrate the median values and their respective ranges. Statistical significance between the groups was determined using a two-tailed Mann-Whitney U test (**p*<0.05, ***p*<0.01, ****p*<0.001, *****p*<0.0001).

## References

[1] Hong GK Payne SC Jane JA Jr. Anatomy, physiology, and laboratory evaluation of the pituitary gland. Otolaryngol Clin N Am. (2016) 49:21–32. doi: 10.1016/j.otc.2015.09.002, PMID: 26614827

[2] Liu X Wang R Li M Chen G. Pituitary adenoma or pituitary neuroendocrine tumor: a narrative review of controversy and perspective. Transl Cancer Res. (2021) 10:1916–20. doi: 10.21037/tcr-20-3446, PMID: 35116513 PMC8798339

[3] Ostrom QT Cioffi G Waite K Kruchko C Barnholtz-Sloan JS. CBTRUS statistical report: primary brain and other central nervous system tumors diagnosed in the United States in 2014-2018. Neuro-Oncology. (2021) 23:iii1. doi: 10.1093/neuonc/noab200, PMID: 34608945 PMC8491279

[4] Roncaroli F Donofrio CA Walker L Laitt R Villa C Majeed W. Update on the classification and diagnostic approach of pituitary neuroendocrine tumours. Diagn Histopathol. (2024) 30:668–79. doi: 10.1016/j.mpdhp.2024.10.001

[5] Villa C. The World Health Organization classifications of pituitary neuroendocrine tumours: a clinico-pathological appraisal. Endocr Relat Cancer. (2023) 30:e230021. doi: 10.1530/ERC-23-0021 37068095

[6] Trouillas J Jaffrain-Rea ML Vasiljevic A Raverot G Roncaroli F Villa C. How to classify the pituitary neuroendocrine tumors (PitNET)s in 2020. Cancers (Basel). (2020) 12:514. doi: 10.3390/cancers12020514, PMID: 32098443 PMC7072139

[7] Sahakian N Appay R Resseguier N Graillon T Piazzola C Laure C. Real-life clinical impact of a five-tiered classification of pituitary tumors. Eur J Endocrinol. (2022) 187:893–904. doi: 10.1530/EJE-22-0812, PMID: 36315463

[8] Cui S. Research status and prospects of pituitary adenomas in conjunction with neurological and psychiatric disorders and the tumor microenvironment, Frontiers in Neuroscience, vol. 18 (2024). 10.3389/fnins.2024.1294417 PMC11075501 38716256

[9] Lang ST Ryu WHA Starreveld YP Costello FE the PITNET Study Group. Good visual outcomes after pituitary tumor surgery are associated with increased visual cortex functional connectivity. J Neuroophthalmol. (2021) 41:504–11. doi: 10.1097/WNO.0000000000001155, PMID: 33399415

[10] Nakaya M. Volumetric measurement of paranasal sinuses and its clinical significance in pituitary neuroendocrine tumors operated using an endoscopic endonasal approach, Frontiers in Neurology. vol. 14 (2023). 10.3389/fneur.2023.1162733 PMC10098075 37064182

[11] Tang H Fang Y Bie Z Jia H Wang B Yang Z. Pituitary neuroendocrine tumor: a neuropsychological comparison with intra-axial tumor. Ann Clin Transl Neurol. (2024) 11:1021–33. doi: 10.1002/acn3.52022, PMID: 38385869 PMC11021612

[12] Manjón JV Romero JE Vivo-Hernando R Rubio G Aparici F de la Iglesia-Vaya M. vol2Brain: a new online pipeline for whole brain MRI analysis. Frontiers in Neuroinformatics. (2022) 16:862805. doi: 10.3389/fninf.2022.862805, PMID: 35685943 PMC9171328

[13] Fischl B Salat DH Busa E Albert M Dieterich M Haselgrove C. Whole brain segmentation: automated labeling of neuroanatomical structures in the human brain. Neuron. (2002) 33:341–55. doi: 10.1016/S0896-6273(02)00569-X 11832223

[14] Fischl B Salat DH van der Kouwe AJW Makris N Ségonne F Quinn BT. Sequence-independent segmentation of magnetic resonance images. NeuroImage. (2004) 23:S69–84. doi: 10.1016/j.neuroimage.2004.07.016, PMID: 15501102

[15] Lin S Li J Wu ZB. Sexual dimorphism in pituitary neuroendocrine tumours. Nature reviews. Endocrinology. (2025) 21:263–4. doi: 10.1038/s41574-025-01096-x, PMID: 40000806

[16] Foundas AL Zipin D Browning CA. Age‐related changes of the insular cortex and lateral ventricles. *Journal of Neuroimaging*. (1998) 8:216–21. 10.1111/jon199884216 9780853

[17] Thomas R Shenoy K Seshadri MS Muliyil J Rao A Paul P. Visual field defects in non-functioning pituitary adenomas. Indian J Ophthalmol. (2002) 50:127–30. PMID: 12194569

[18] Abouaf L Vighetto A Lebas M. Neuro-ophthalmologic exploration in non-functioning pituitary adenoma. Ann Endocrinol (Paris). (2015) 76:210–9. doi: 10.1016/j.ando.2015.04.006, PMID: 26070465

[19] Ferrante E Ferraroni M Castrignanò T Menicatti L Anagni M Reimondo G. Non-functioning pituitary adenoma database: a useful resource to improve the clinical management of pituitary tumors. Eur J Endocrinol. (2006) 155:823–9. doi: 10.1530/eje.1.02298, PMID: 17132751

[20] Esposito D Olsson DS Ragnarsson O Buchfelder M Skoglund T Johannsson G. Non-functioning pituitary adenomas: indications for pituitary surgery and post-surgical management. Pituitary. (2019) 22:422–34. doi: 10.1007/s11102-019-00960-0, PMID: 31011999 PMC6647426

[21] Jaffe CA. Clinically non-functioning pituitary adenoma. Pituitary. (2006) 9:317–21. doi: 10.1007/s11102-006-0412-9, PMID: 17082898

[22] Ntali G Wass JA. Epidemiology, clinical presentation and diagnosis of non-functioning pituitary adenomas. Pituitary. (2018) 21:111–8. doi: 10.1007/s11102-018-0869-3, PMID: 29368293

[23] Rizzoli P Iuliano S Weizenbaum E Laws E. Headache in patients with pituitary lesions: a longitudinal cohort study. Neurosurgery. (2016) 78:316–23. doi: 10.1227/NEU.0000000000001067, PMID: 26485333

[24] Catani M Dell’Acqua F Thiebaut de Schotten M. A revised limbic system model for memory, emotion and behaviour. Neuroscience and Biobehavioral Reviews. (2013) 37:1724–37. doi: 10.1016/j.neubiorev.2013.07.001, PMID: 23850593

[25] Duvernoy HM. The human hippocampus: An atlas of applied anatomy. Munich: JF Bergmann-Verlag (2013).

[26] Naidich TP. Duvernoy's atlas of the human brain stem and cerebellum: High-field MRI, surface anatomy, internal structure, vascularization and 3 D sectional anatomy. Vienna: Springer Science & Business Media (2009).

[27] Ge Y-J Wu BS Zhang Y Chen SD Zhang YR Kang JJ. Genetic architectures of cerebral ventricles and their overlap with neuropsychiatric traits. Nat Hum Behav. (2024) 8:164–80. doi: 10.1038/s41562-023-01722-6, PMID: 37857874

[28] Arakaki X Butler R Kang J Macias T Chui HC Nolty A. MRI norm percentile in age‐related atrophy starts from MCI stage. Alzheimer’s and Dementia. (2022) 18:e064364. doi: 10.1002/alz.064364, PMID: 40843626

[29] Park HK Marston L Mukadam N. The effects of estrogen on the risk of developing dementia: a cohort study using the UK biobank data. Am J Geriatr Psychiatry. (2024) 32:792–805. doi: 10.1016/j.jagp.2024.01.025, PMID: 38310026

[30] Pourhadi N Mørch LS Holm EA Torp-Pedersen C Meaidi A. Menopausal hormone therapy and dementia. William Babington. (2023) 381:e072770. doi: 10.1136/bmj-2022-072770, PMID: 37380194 PMC10302215

[31] Janicki SC Schupf N. Hormonal influences on cognition and risk for Alzheimer's disease. Curr Neurol Neurosci Rep. (2010) 10:359–66. doi: 10.1007/s11910-010-0122-6, PMID: 20535591 PMC3058507

[32] Cui S Chen S Wu X Wang Q. Research status and prospects of pituitary adenomas in conjunction with neurological and psychiatric disorders and the tumor microenvironment. Frontiers in Neuroscience. (2024) 18. doi: 10.3389/fnins.2024.1294417, PMID: 38716256 PMC11075501

[33] Tirosh A Papadakis GZ Chittiboina P Lyssikatos C Belyavskaya E Keil M. 3D volumetric measurements of GH secreting adenomas correlate with baseline pituitary function, initial surgery success rate, and disease control. Horm Metab Res. (2017) 49:440–5. doi: 10.1055/s-0043-107245, PMID: 28472827 PMC6309337

[34] Gundogdu DK Gezer B Sahinoglu M Koktekir E Karabagli H Bozkurt MA. Impact of tumor resection volume on visual outcomes and the need for secondary surgery following Transsphenoidal surgery in Suprasellar extended non-Functionial pituitary adenomas. Turk Neurosurg. (2024) 34:991–8. doi: 10.5137/1019-5149.JTN.45315-23.2, PMID: 39474971

[35] Ahmadi J North CM Segall HD Zee CS Weiss MH. Cavernous sinus invasion by pituitary adenomas. AJR Am J Roentgenol. (1986) 146:257–62. 3484572 10.2214/ajr.146.2.257

[36] Burlacu MC Maiter D Duprez T Delgrange E. T2-weighted magnetic resonance imaging characterization of prolactinomas and association with their response to dopamine agonists. Endocrine. (2019) 63:323–31. doi: 10.1007/s12020-018-1765-3, PMID: 30267354

[37] Yu YL Yang YJ Lin C Hsieh CC Li CZ Feng SW. Analysis of volumetric response of pituitary adenomas receiving adjuvant CyberKnife stereotactic radiosurgery with the application of an exponential fitting model. Medicine (Baltimore). (2017) 96:e4662. doi: 10.1097/MD.0000000000004662, PMID: 28121913 PMC5287937

[38] Galland F Vantyghem MC Cazabat L Boulin A Cotton F Bonneville JF. Management of nonfunctioning pituitary incidentaloma. Ann Endocrinol (Paris). (2015) 76:191–200. doi: 10.1016/j.ando.2015.04.004, PMID: 26054868

[39] Harary M DiRisio AC Dawood HY Kim J Lamba N Cho CH. Endocrine function and gland volume after endoscopic transsphenoidal surgery for nonfunctional pituitary macroadenomas. J Neurosurg. (2019) 131:1142–51. doi: 10.3171/2018.5.JNS181054, PMID: 30497144

[40] Jack CR Jr Bernstein MA Fox NC Thompson P Alexander G Harvey D. The Alzheimer's disease neuroimaging initiative (ADNI): MRI methods. J Magn Reson Imaging. (2008) 27:685–91. doi: 10.1002/jmri.21049, PMID: 18302232 PMC2544629

[41] Tóth A Schmalfuss I Heaton SC Gabrielli A Hannay HJ Papa L. Lateral ventricle volume asymmetry predicts midline shift in severe traumatic brain injury. J Neurotrauma. (2015) 32:1307–11. doi: 10.1089/neu.2014.3696, PMID: 25752227 PMC4545563

[42] Bulekbayeva B., Pediyatrik olgularda hidrosefali tanısında ve tedavi sonuçlarının değerlendirilmesinde radyolojik görüntülemenin katkısı. İzmir: Ege University (2013).

[43] Kang K Kwak K Yoon U Lee JM. Lateral ventricle enlargement and cortical thinning in idiopathic Normal-pressure hydrocephalus patients. Sci Rep. (2018) 8:13306. doi: 10.1038/s41598-018-31399-1, PMID: 30190599 PMC6127145

