# Supplementary figures and images for: The effect of pituitary neuroendocrine tumors on the volumes of intracranial structures
Source: Front Neurol. 2025 Sep 10;16:1585921. doi: 10.3389/fneur.2025.1585921 (PMC12457147; doi:10.3389/fneur.2025.1585921)

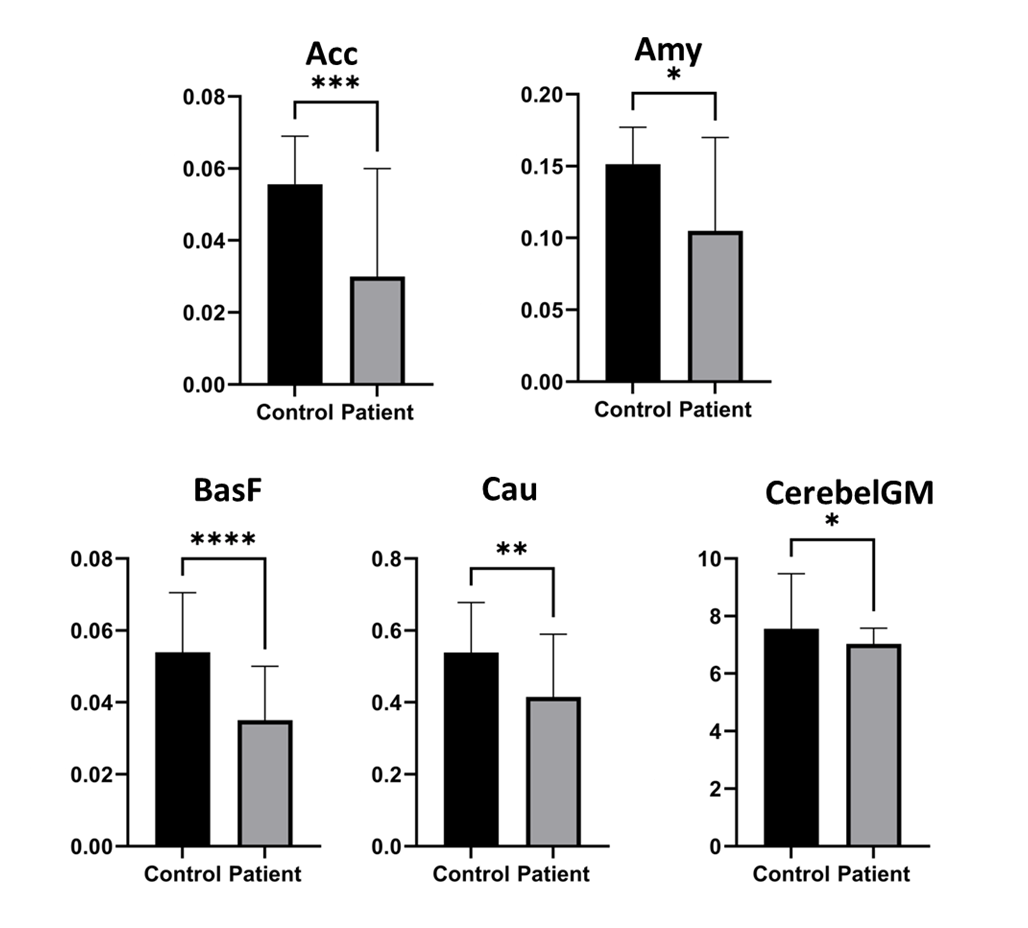

Supplement: SUPPLEMENTARY FIGURE 1 — Analysis of total basal nuclei and cerebellar gray matter volumes. Acc, Nucleus accumbens, Amy, Amygdala, BasF, Basal forebrain, Cau, Nucleus caudatus, and CerebelGM, Cerebellar gray matter volume. Bar plots illustrate the median values and their respective ranges. Statistical significance between the groups was determined using a two-tailed Mann-Whitney U test (*p<0.05, **p<0.01, ***p<0.001, ****p<0.0001). [file Image_1.TIF]

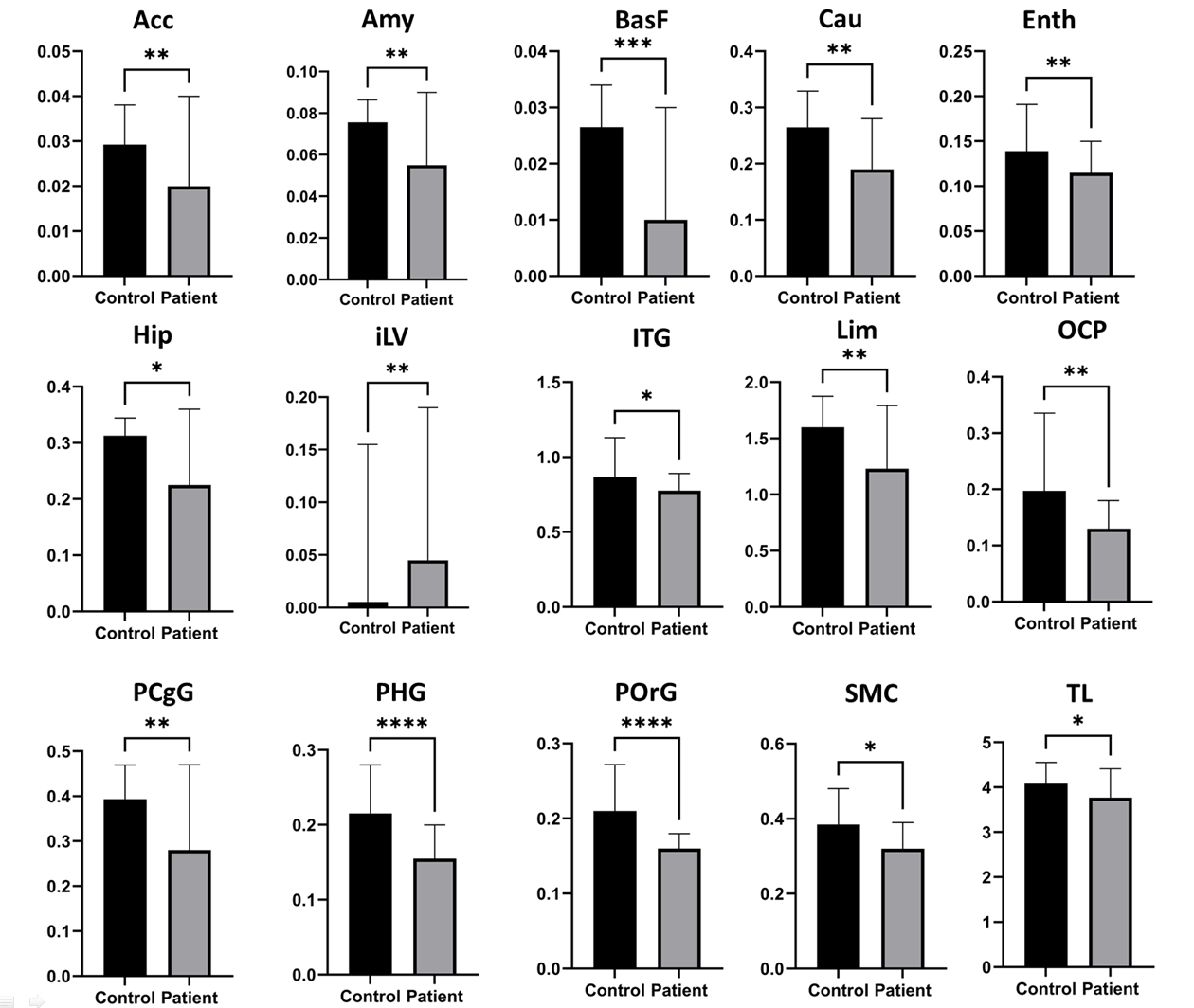

Supplement: SUPPLEMENTARY FIGURE 2 — Volumetric difference analysis results of structures located in the left hemisphere between PitNET and healthy control groups. Acc, Nucleus accumbens, Amy, Amygdala, BasF, Basal forebrain, Cau, Nucleus caudatus, Enth, Entorhinal area, Hip, Hippocampus, iLV, Inferior lateral ventricle, ITG, Inferior temporal gyrus, Lim, Limbic lobe, OCP, Occipital pole, PCgG, Posterior cingulate gyrus, PHG, Parahippocampal gyrus, POrG, Posterior orbital gyrus, SMC, Supplementary motor cortex, TL, Temporal lobe. Bar plots illustrate the median values and their respective ranges. Statistical significance between the groups was determined using a two-tailed Mann-Whitney U test (*p<0.05, **p<0.01, ***p<0.001, ****p<0.0001). [file Image_2.TIF]

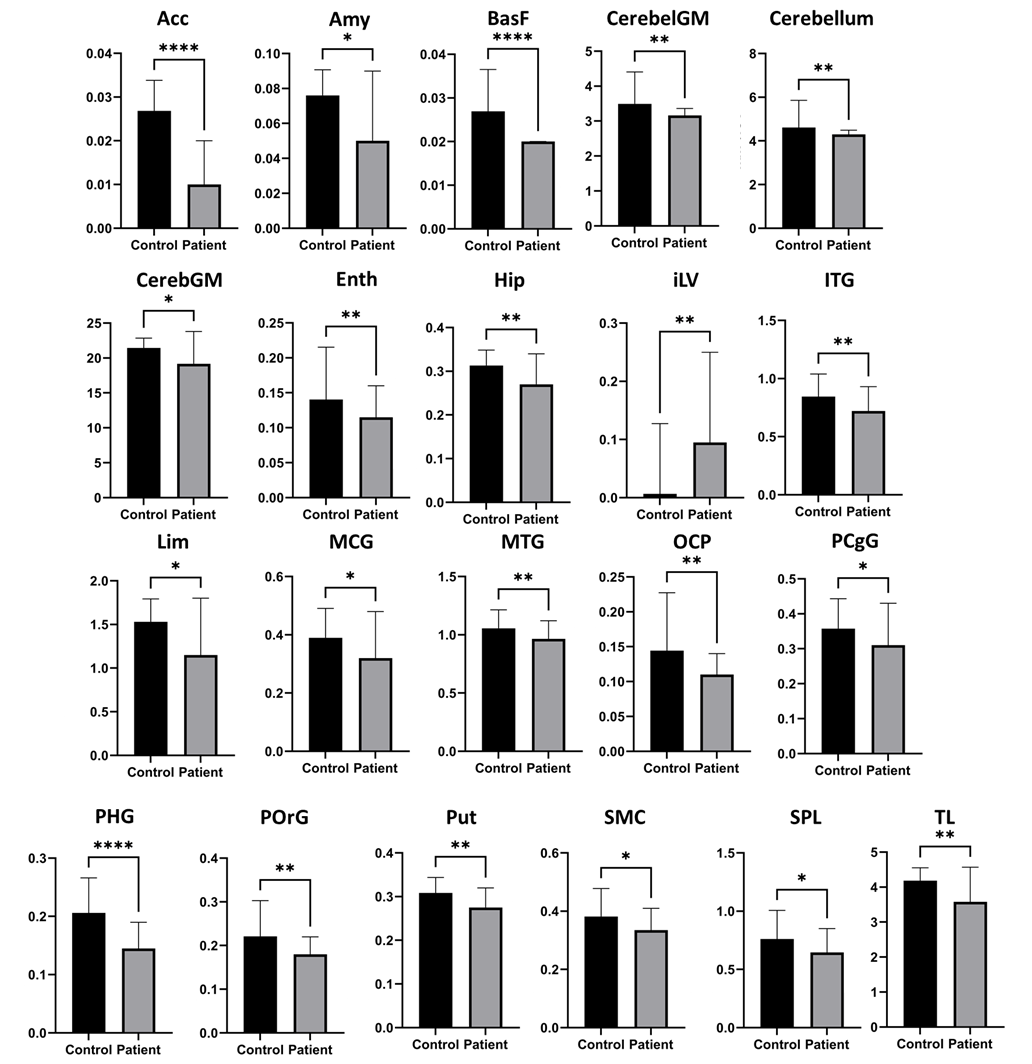

Supplement: SUPPLEMENTARY FIGURE 3 — Volumetric difference analysis results of structures located in the left hemisphere between PitNET and healthy control groups. Acc, Nucleus accumbens, Amy, Amygdala, BasF, Basal forebrain, CerebelGM, Cerebellar GM volume, Cerebellum, Left Cerebellum Hemisphere, Enth, Entorhinal area, Hip, Hippocampus, iLV, Inferior lateral ventricle, ITG, Inferior temporal gyrus, Lim, Limbic lobe, MCG, Middle cingulate gyrus, MTG, Middle temporal gyrus, OCP, Occipital pole, PCgG, Posterior cingulate gyrus, PHG, Parahippocampal gyrus, POrG, Posterior orbital gyrus, Put, Putamen, SMC, Supplementary motor cortex, SPL, Superior Parietal Lobule, TL, Temporal lobe. Bar plots illustrate the median values and their respective ranges. Statistical significance between the groups was determined using a two-tailed Mann-Whitney U test (*p<0.05, **p<0.01, ***p<0.001, ****p<0.0001). [file Image_3.TIF]
